# Comparison of basic motor skills and physical fitness between (pre-)pubertal children from parkour and team sports

**DOI:** 10.3389/fspor.2025.1562561

**Published:** 2025-03-27

**Authors:** Chris Konushevci, Joel Mason, Konstantin Warneke, Astrid Zech

**Affiliations:** ^1^ Department of Sport Science, University of Vechta, Vechta, Germany; ^2^ Department of Human Movement Science and Exercise Physiology, Institute of Sport Science, Friedrich Schiller University Jena, Jena, Germany

**Keywords:** adolescents, children, jumping, parkour, physical fitness, plyometrics, skills, team sports

## Abstract

**Background:**

Parkour is a modern sport known for daring jumps and moves in urban environments that require exceptional motor skills and various sports-specific techniques. Although it is increasingly popular among children and adolescents, training routines in youth Parkour are still rather driven by personal beliefs and experience of coaches than by evidence.

**Purpose:**

This study aims to analyze basic motor skills and physical fitness of youth Parkour athletes compared to team sports athletes.

**Study design:**

Cross-sectional study with matched-pair analysis.

**Methods:**

Seventeen youth Parkour (12.50 ± 1.80 years) and seventeen team sports athletes (11.90 ± 1.70 years), matched for height and weight, participated in this study. Tests included static (single-leg postural sway = PS) and dynamic balance (Y-Balance test = YBT), jumping (countermovement jump = CMJ, drop jump = DJ, side-hop = SH), muscle strength (planks, pull-ups = PU) and basic gymnastics skills (bridging = BG, handstand = HS, cartwheel = CW).

**Results:**

The Parkour group performed significantly better in the CMJ (***p*** **=** **0.014**), the anterior direction of the YBT (***p*** **<** **0.001**), cartwheel performance (***p*** **=** **0.019**), and pull-ups (***p*** **=** **0.029**) when compared to the team-sports group. Moderate but non-significant differences were observed in PS for the dominant (*p* = 0.12) and non-dominant leg (*p* = 0.14) as well as in SH (*p* = 0.06). No further significant differences were observed.

**Conclusion:**

Children practicing Parkour demonstrated superior performances in certain parameters of motor skills and physical fitness compared to team sports athletes. The findings suggest that Parkour may contribute positively to children's overall physical development. However, more intervention studies with a prospective study design are needed for further recommendations.

## 1 Introduction

Parkour, a rapidly growing sport among youth, originated in late-20th century France, evolving from Georges Hébert's military training method, *la méthode naturelle* (1–3). Rooted in functional movements like running, climbing, and jumping (4), it was later adapted by David Belle into an urban discipline in the 1980s–90s (3, 5). While research on its social and psychological aspects is expanding, studies on its physiological impact, particularly in youth athletes, remain limited (6, 7).

Opposed to Parkour, team sports such as football (soccer), rugby, basketball, volleyball or handball are well-established and remain highly popular across all ages. Required skills include high-intensity and often asymmetric plyometric actions or maneuvers such as sprints, jumps or changes of direction (8) as well as a high degree of general physical fitness (9, 10). The main objectives of jumping maneuvers in these sports are both defensive (e.g., blocking, rebounding or ball-catching) as well as offensive (e.g., throwing, hitting or kicking a ball) with unilateral and bilateral plyometric actions (11, 12).

It has already been hypothesized that team sports and Parkour require similar fundamental cognitive (e.g., finding creative solutions for problem situations and decision making as well as) and motor (e.g., rapid change of direction and speed, jumping and landing) skills (13). Although team sports athletes’ (TSA) (for all abbreviations see Table 1) jumping movement patterns are complex and sport-specific, there are overlaps in movement profiles and physiological key parameters with Parkour. For instance, in both sports, plyometric actions such as jumping and sprinting require the ability to produce force in a minimal time window while storing and releasing energy in the muscle tendon complex. When focusing on extremely short (commonly <0.25 s) the short stretch-shortening cycle (SSC) gets dominant, while for strength dominant jumps the slow SSC is focused (14). Here, vertical jumps across all domains typically consist of a visible countermovement parameter and can be observed as a central element in Parkour specific movement (5, 15). The same holds true for soccer (16), volleyball (17), basketball (18) as well as handball (19) athletes and, therefore, undeniably correlates with performance (12, 20). Additionally, researchers have analyzed both health-related (11, 21, 22) and skill-related components (8, 23, 24) in TSA while recent studies have also shown enhanced physical fitness and motor skills in adult traceurs (5, 25, 26). To our knowledge, only three studies focused on children, exploring the feasibility of Parkour in primary school (27), effects on motor skill development and transfer (28), and the individual motives for practicing Parkour (29). No study has analyzed its benefits on physical fitness and motor skills compared to other popular sports. By comparing Parkour to team sports, potential gaps or overlaps in benefits could be identified, allowing athletes to cross-train effectively by selecting complementary activities (13). Due to the described similarities in motor skills between the sports, this study aims to compare motor performance between TSA and Parkour athletes. It is hypothesized that children who regularly practice Parkour will demonstrate similar or superior performance in measurable motor skills and physical fitness parameters, specifically in jumping, balance, muscle strength, and basic gymnastics skills, compared to children participating in team sports. To avoid potential ceiling effects of standardized tests in individuals with advanced motor skills, additional gymnastics basics such as handstand or cartwheel were measured (30). The findings will help to understand the potential of regular Parkour training for the motor development of children and adolescents.

**Table 1:** List of abbreviations.

Abbreviation	Meaning
BG	Bridging
BMI	Body mass index
CMJ	Countermovement jump
COD	Change of direction
CW	Cartwheel
DJ	Drop jump
GCT	Ground contact time
HS	Handstand
PG	Parkour group
PS	Postural sway
*d*	Dominant (leg)
*nd*	Non-dominant (leg)
PU	Pull-ups
*wins*	Winsorization
ROM	Range of motion
SH	Side hops
SSC	Stretch-shortening cycle
TSA	Team sports athletes
TG	Team sports group
YBT	Y-balance test
*ant*	Anterior
*pm*	Posterior medial
*pl*	Posterior lateral

## 2 Materials and methods

### 2.1 Participants

Parkour youth athletes (PG) were recruited from a local Parkour sports club, while team sports athletes (TG) were recruited from local youth soccer, volleyball, and handball teams. A two-stage sampling approach was used. The PG was randomly selected based on predefined criteria. These inclusion criteria were: an age range between 9 and 15 years, attending training and competitions regularly (at least twice per week), having at least three months of sports-specific experience as well as being free of current injuries. Ankle injuries occurring within three months prior to testing as well as any severe injury that could influence the performance were exclusion criteria. Children were excluded if they were currently active in both sports (Parkour and team sports) as including children with mixed training backgrounds could blur the differences. However, previous experience through any possible contact with the other sport (e.g., during sport education in school) was not prohibited. The TG was matched to the first group based on the same criteria, with additional pairwise matching for height and weight. Ethical approval was obtained from the local ethics committee (FSV 22/082). The athletes as well as their legal guardians were informed about the risks and benefits of the study and gave written informed consent.

### 2.2 Tests and procedures

The test battery included standardized tests of physical fitness and motor skills (31) (Supplementary Figure S1). On the first test day jumping and balance skills were tested using a portable 3D force plate (Kistler, Germany, Type 9260AA). All other tests were performed about one week (5–7 days) after the first testing procedure. The test order was the same for all participants to keep fatigue as minimal as possible. Participants were allowed to rest for at least one minute between tests. Participants were also familiarized with test procedures to avoid learning effects.

#### 2.2.1 Jumping tests

In order to perform a countermovement jump (CMJ), participants were asked to bend their knees at an angle of approximately 90° and to jump in an explosive manner as high as possible (32). Participants were given three trials in total with a one-minute recovery interval in-between. A trial was considered invalid and repeated if the participant landed partially on or next to the force plate, removed their arms during the CMJ, or failed to keep their legs straight while airborne.

Further, subjects were asked to perform a drop jump (DJ) from a box of 40 cm height with hands placed on their hips. Immediately after landing, the participants performed a bilateral maximum vertical jump with their arms resting tightly on their hips. The legs needed to remain straight in mid-air. Three trials were given. The focus of the DJ was to assess the reactive force skills. In order to achieve that goal with simple instructions we asked the children to jump as fast as possible after ground contact (not to jump as high as possible) while not just bouncing.

For the bilateral side hops (SH) test two strips of tape were placed on the floor with a distance of 40 cm (33). Participants were instructed to place their hands on their hips and jump side to side as many times as possible within 15 s without touching the tape or moving their hands from their hips. If rhythm was lost, the children were encouraged to continue as fast as possible. A camera was placed in front of them and used to ensure the correct number of ground contacts. Two attempts were conducted with a break of 60 s between these. The best attempt was used for statistical analysis. All tests are established, valid and reliable for assessing jumping ability in children and young adults (33).

#### 2.2.2 Balance

Static balance or postural sway (PS) was assessed using center of pressure displacement during a single-leg stance on the dominant leg (*d*) on the force plate. Leg dominance was identified by asking for the jumping leg. During the single-leg stance, participants were asked to stand as motionless as possible with eyes on a target in front and head in a neutral as well as resting their hands on their hips. Data were collected for 30s during quiet standing. An attempt was invalid if balance was lost, entirely, or when the hands left the hips in order to regain control. After another 30 s, the non-dominant leg (*nd*) was assessed. PS is a commonly used and therefore reliable measure of single-leg balance capabilities (23).

For the Y-Balance-Test (YBT), participants were asked to stand upright with their hands on their hips on their dominant leg and to move a wooden distance indicator with the non-standing leg as far as possible into three directions (anterior, posterolateral, posteromedial) (33). After each trial the non-balancing leg returned to the starting position without losing balance. An invalid trial was discarded and another additional trial was completed. It was then emphasized that it was not a competition and participants should only aim for the distance they are capable of in order to avoid an abundance of attempts and thus learning effects. Participants were allowed to familiarize with the single leg stance and proprioceptive demands. This procedure ensured that all participants were treated equally. Criteria for invalid results were lifting the heel of the balancing leg, touching the ground before the end of the trial with the non-balancing leg, lifting hands from hips and kicking the sliding element or placing weight onto it. Thereafter, the non-dominant leg was tested. An average of the two trials was calculated for each leg. Reach distances were adjusted for leg length. The YBT is one of the most frequently used assessments for lower limb function and, when performed under standardized conditions across all groups, one of the most reliable tests for assessing group differences as studies have shown (33).

#### 2.2.3 Muscle strength

Planking is a common fitness exercise and assessment method for core strength for adults as well as children (34). The maximum time was set at 2:30 min. A proper plank position was defined by a clear scapula protraction and maintaining a straight line from head to heels throughout the event. Due to maximum exhaustion and prolonged recovery, participants were given only one attempt.

Pull-ups (PU) on a horizontal immovable overhead bar was used to assess upper body strength. Boys had no ground contact during the entire exercise while girls kept their feet, with straight legs, on the ground. The shoulders, hands, and bar were aligned vertically, with the hands in a pronated grip and positioned approximately shoulder-width apart. Participants were instructed to pull up while keeping the core in position and legs as motionless as possible. A pull-up was counted when the shoulder angle reached approximately 45° with the chin above the bar during the concentric phase, and the elbows were fully locked at the end of the eccentric phase, without the use of momentum. Due to maximum exhaustion only one attempt was given. It is a well-established and widely used test for assessing upper body strength, particularly in children (31).

#### 2.2.4 Gymnastics skills

This test battery comprised bridging, handstand, and cartwheel. These fundamental gymnastics skills require strength, coordination, flexibility, and balance, making them valuable indicators of skill-related physical fitness (35), particularly in the context of avoiding ceiling effects (30). Since they lack standardized criteria (36), rating them is challenging, especially in a study not focused on gymnastic perfection. While certain success criteria exist, they vary between elements, particularly for dynamic movements, and are less clearly defined for static elements like bridging. Therefore, ratings were categorized into yes (completely fulfilled) and no (unsuccessful). In order to ensure robust evaluation and meet prescribed joint ankles an app named Coach's Eye (TechSmith Corporation, Okemos, MI, USA) was used (37). Furthermore, the observer is a licensed gymnastics coach with a highly trained eye for assessing these basic gymnastics tests, which helps minimize subjectivity in the evaluation process. Files were imported and analyzed according to the criteria below. Before each test, participants were given time for an unspecific warm-up.

##### 2.2.4.1 Bridging

Bridging (BG) is considered to be a gymnastics basic skill and portrays the gymnast's level of static flexibility of the shoulders and dorsal extension capacities of the spine (38). Although it is a frequently used test for gymnastics assessment there is no standardized procedure (39). The study used a slightly altered version of the protocol described by Vernetta et al. in their 2017 publication (38). While lying on the floor, the athletes had to place their hands behind their head. They were told to push themselves away from the ground into a parabolic position. Athletes were further instructed to straighten their arms and legs while pushing their body toward their shoulders. To be acceptable, the BG needed to be held for five seconds with a knee angle of at least 100° and shoulders aligned with hands (±20°). A camera was placed perpendicular to the athlete in order to assess the angles. This upper angle had to be ≤80°. The camera was placed perpendicular to the athlete with a distance of five meters. One attempt was given.

##### 2.2.4.2 Handstand

Handstand (HS) is another fundamental gymnastics skill (40). A soft mat was placed in front of the standing athlete for safety. They were free to enter and leave the HS in their desired way. A camera was placed perpendicular to the athlete in order to capture the posture upside-down. Participants were given three consecutive attempts. A successful (‘yes’) attempt included a shoulder angle of about >150° but not smaller, straight hips (180° ± 20°) aligned with the shoulders and feet. Knees had to be nearly straight with minimum angle of 160°.

##### 2.2.4.3 Cartwheel

The cartwheel (CW) was performed on a straight line on the floor. Determinants for a successful execution were an open shoulder (≥140°) and knee angle (≥160). Three attempts were given for each participant. The favored side could be chosen. Attempts were considered successful as soon as one out of three attempts was valid.

## 3 Statistical analysis

Statistics were computed using ‘Paleontological Statistics’. T-tests were employed to compare height and weight between matched groups. One-way ‘Analysis of Variance’ (ANOVA) was employed to probe potential group differences for the variables ‘experience’ and ‘age’. All data were checked for normal distribution and homogeneity of variances. A confidence interval of 95% (*CI* *=* *95%*) was adopted in advance. The effect size ‘partial eta-squared’ ($\eta_{p}^{2}$) was computed to assess the magnitude of group differences. To complement the ANOVA, ‘Pearson's Chi-square’ (*χ*^2^) contingency analysis was implemented in order to assess associations between categorical data on successful and unsuccessful gymnastics assessments. If results were susceptible to outliers, a mitigation technique (winsorization) was employed to reduce the impact. These were addressed by replacing the upper outliers with the 95th percentile and lower outliers with the 5th percentile. Pearson's correlation coefficient (*r*) was implemented for jumping outcomes.

## 4 Results

In total, 34 healthy youth athletes participated in this study. One participant was unable to attend the second test day. Descriptive statistics are displayed in Table 2. No statistically significant group differences were found for the matched variables weight (*p* = 0.96) and height (*p* = 0.99), or for the unmatched variable age (*p* = 0.33).

**Table 2:** Participants’ descriptive data (mean ± SD).

Variable	PG		TG	
	Gender			
	Women	Men	Women	Men
	2	15	2	15
Age (years)	12.50 ± 1.80		11.90 ± 1.70	
Height (meters)	1.60 ± 0.14		1.60 ± 0.14	
Weight (kg)	47.30 ± 12.80		47.10 ± 11.80	
BMI (kg/m^2^)	17.97 ± 2.41		17.96 ± 1.96	
Experience (months)	13.00 ± 9.80		46.50 ± 25.00	
Weekly training (days)	2.00 ± 0.00		3.00 ± 1.37	

### 4.1 Jumping

The CMJ performance was significantly (***p*** **=** **0.014**) different between groups with favorable results for the PG (Table 3). No significant groups differences were found for DJ (*p* = 0.52) performance. Although the PG demonstrated a considerably higher number of side hops compared to the TG, the group difference was non-significant (*p* = 0.06). The correlation analyses between CMJ and SH as well as DJ and SH indicate a weak positive (*r* = 0.21) as well as moderate positive (*r* = 0.41) relationship in the PG, respectively. In the TG, there is a moderate (*r* = 0.45) positive correlation between CMJ and SH, whereas there is a moderate (*r* = −0.40) negative correlation between DJ and SH, simultaneously.

**Table 3:** Statistical indicators (testing).

Tests	Variable	TG		PG		Statistics			
		Mean	SD	Mean	SD	F	95% CI	*p*-value	$\eta_{p}^{2}$
Jumping	CMJ (cm)	0.26	0.05	0.31	0.06	6.81	(0.01, 0.09)	0.014	0.18
	DJ (s)	0.24	0.04	0.25	0.07	0.43	(−0.03, 0.05)	0.52	0.01
	SH (score)	28.44	3.65	30.82	3.76	3.67	(−0.16, 5.31)	0.06	0.11
Balance	PS d (mm/s)	54.02	19.08	44.11	16.56	2.65	(−2.57, 22.39)	0.12	0.08
	PS nd (mm/s)	53.22	15.71	45.15	15.34	2.30	(−2.78, 18.91)	0.14	0.07
	YBT ant (cm)	56.40	6.72	66.95	4.43	55.84	(7.76, 13.33)	<0.001	0.47
	YBT pm (cm)	100.19	7.60	98.06	7.28	0.19	(−3.77, 5.85)	0.67	0.00
	YBT pl (cm)	90.27	10.00	91.31	9.57	1.36	(−1.52, 5.80)	0.25	0.02
Muscle Strength	Plank (s)	126.40	26.00	116.40	38.30	0.76	(−13.38, 33.43)	0.39	0.02
	PU (score)	2.94	3.97	5.47	4.13	3.22	(−0.35, 5.41)	0.08	0.09
	PU wins	–	–	–	–	5.20	(0.09, 5.44)	0.029	0.14
Tests	Variable	Success	Fail	Success	Fail			*p*-value	*χ* ^2^
Gymnastics skills	BG	7	9	11	6			0.23	1.46
	HS	0	16	0	17			1.00	0.00
	CW	3	13	10	7			0.019	5.54

### 4.2 Balance

Postural sway on the dominant (*p* = 0.12) and non-dominant (*p* = 0.14) leg were not significantly different between groups. In contrast, YBT anterior reach distance was significantly higher (***p* < 0.001**) in the PG compared to the TG while no significant differences were found for posterior reach directions (Table 3).

### 4.3 Muscle strength

No significant group differences were found for planking duration (*p* = 0.39) or number of PUs (*p* = 0.08). After adjusting for outliers (Supplementary Figure S2) by winsorization technique a statistically significant difference (***p*** **=** **0.029**) was found between groups with favorable results in the PG.

### 4.4 Gymnastics skills

Chi-square tests revealed that there are no significant group differences in BG (*p* = 0.23) and HS (*p* = 1.00). However, a significant difference was found in performing a CW (***p*** **=** **0.019**). Whereas only three were able to perform a correct CW in the TG, ten PG athletes were successful.

## 5 Discussion

Our key findings suggest that regular Parkour training can be more beneficial for children's and adolescents’ athletic and general gymnastics skills than training in team sports. In each tested performance or skill category (jumping, balance, strength and gymnastics skills) traceurs showed either similar or better performances than matched team sport athletes. This agrees with our main hypothesis and emphasized the great potential of Parkour for motor development, either as primary or as secondary sport.

### 5.1 Jumping performances

Jump abilities are fundamental for performance in both sports. In order to address the different dimensions of jumping, assessments included maximum vertical jump height during the CMJ, ground contact time in the DJ test as an indicator of reactive strength, and the number of side hops (SH) in a 15s trial. Only for the CMJ significant differences were observed between team sports and Parkour athletes. The superior performance of the PG indicates that the power generated by the SSC during the countermovement jump is more developed in traceurs. Skills like bouncing, leaping and jumping are fundamental movements of Parkour and may thus be responsible for advanced explosive jumping skills (5). As for DJ, the TG has been shown to have slightly shorter ground reaction times. This result was not unexpected considering that traceurs may be used to precision during jumping chains whereas the DJ is more unfamiliar and therefore in conflict with habitual landing techniques, specifically in the amortization phase after a chain of jumps (4, 41, 42). You & Huang hypothesize “that drop jump stiffness could be altered by different landing technique” (43). Stiffer jumps would result in better reactive strength whereas softer jumps are likely to be measurably higher (43). This agrees with our findings and may explain the differences between CMJ and DJ. It is suggested that short ground contacts (<0.25 s) are particularly important for sprinting velocity (44). Freerunning, as an acrobatic version of Parkour (3, 6, 45), typically focuses on flow and includes diverse and creative gymnastics movements such as somersaults (45). It has been proven that gymnasts have extremely short GCTs which allows them to reach considerable heights in their floor routines (46). However, since no associations between specific Parkour techniques and performance were tested in this study, future research is needed to analyze the potential impact of Freerunning and the development of reactive and explosive strength. Nonetheless, due to a versatile jumping repertoire, Parkour may be seen as a different form of plyometric training (PT), as current research indicates (5). Hence, based on the findings of this study and of previous research it seems reasonable to expect positive effects of Parkour training on SSC dynamics.

Furthermore, the observed correlation between DJ and SH in the Parkour group suggests that poorer DJ performance is associated with better SH performance. Although the team sports group exhibited moderate positive correlations between CMJ and SH and moderate negative correlations between DJ with SH, suggesting that both higher and faster jumps were associated with better bilateral side hopping results, these findings remain inconclusive and speculative. A potential CMJ-SH correlation indicates an unusual transfer from slow SSC to SH performance, which is unexpected due to SH's reliance on leg stiffness (47, 48). The small sample size may also contribute to these uncertainties. The potential transfer of CMJ or DJ performance to bilateral SH was the only interesting area since single-leg lateral jumps have been shown to be a predictor of agility, change of direction (COD) and sprinting (49). To better understand the SSC's role as the underlying mechanism across these jumping tests as well as to explore sport-independent inter-test correlations, further research is needed.

### 5.2 Balance

Our findings indicate that both team sport athletes and traceurs have a comparable level of static and dynamic balance skills. Postural sway and YBT results were expected to be more pronounced in traceurs since their landing pattern and the action of sticking a jump demands a high degree of control and balance, especially when they land precisely on rails (5). In 2018, Maldonado showed that the anterior direction seems specifically important when executing soft precision landings at the end of jumps or plyometric jumping chains (41). This was confirmed by the PG's significantly better performance in the anterior direction of the YBT*.* This could be due to more active ROM (dorsiflexion during ankle regulation in mid-air) combined with passive ROM (varying ankle angles) as well as enhanced control during eccentrics (5, 26, 41). A similar pattern may explain the slightly enhanced performance of the TG in YBT in the posterior medial direction*.* Balance is essential for kicking actions (23) that are crucial for football (soccer) performance. Furthermore, the YBT is often used in young and physically active people in order to rate sensorimotor control or injury risk (50). In 2018, John et al. have shown that in particular the YBT anterior reach distance responds sensitive to challenging phases of growth, such as peak height velocity (50). Other authors have highlighted the predictive value of the anterior YBT for the lower extremity injury risk (51). Our study seems to support the assumption that the YBT anterior score is a sensitive parameter for discriminating balance performance in young athletes. Furthermore, based on the results, it can be speculated that Parkour has a positive effect on the growth-related motor changes and the risk of injury in adolescents (25). However, conclusive statements cannot be made, since there is still only a limited number of studies on this subject.

### 5.3 Muscle strength

Our findings regarding the PU test also indicate that traceurs possess significantly greater upper body strength compared to team sport athletes. No significant differences in core strength were found between groups, based on the plank position test. Whether these strength skills are more developed than in other sports or compared to no sports has not been tested in our study. However, based on the findings of Dvorak et al. in 2017, it is expected that Parkour training is beneficial for improving core muscle strength as well as general upper body strength (25). Based on PU assessments, our study supports this finding. It could be speculated that Parkour, when incorporated into cross-sport training (13) or implemented in school PE fitness programs focusing on upper body strength, could promote even greater strength gains than the team sports analyzed. Given its engaging nature, Parkour may serve as an appealing alternative to traditional team sports, particularly for children who do not enjoy competitive sports (27, 52).

### 5.4 Gymnastics skills

Participants of both groups were unable to perform the required HS technique. Although we considered the HS a basic gymnastics skill it appears to be a highly specific motor skill within gymnastics that may require exclusive practice of the task. Another explanation could be the young age of participants and, therefore, limitations in muscle strength abilities. The majority of athletes of both sports were able to perform a gymnastics bridge. However, it remains speculative whether this is due to enhanced physical activity in general or related to sports-specific exercising in both sports. The significantly better CW test results and improved BG performance suggest that children practicing Parkour may have an initial advantage when acquiring these skills. This could be addressed in future research, as only three studies have so far explicitly examined Parkour and gymnastics within the same context (2, 53, 54).

## 6 Limitations

One limitation of this study is the disparity in sport-specific experience between groups, which significantly impacts result interpretation, as a less experienced group is unlikely to outperform a more experienced one. While this underscores Parkour's low-threshold accessibility, matching groups would have mitigated this confounding factor. However, the sport's youth, the typical traceur's age (2), and the limited timeframe of the study made achieving this matching practically unfeasible. Children with experience in both sports were deliberately excluded, allowing for a more reliable assessment of sport-specific effects. If participants had experience in both sports, it would be difficult to attribute performance differences to one specific sport since motor skills and physical fitness could be influenced by training adaptations from both disciplines. This would reduce the internal validity of the findings. Future research could explore cross-sport influences separately with a dedicated study design. Another limitation is the small sample size that does not fully represent the broader population of young Parkour and team sports athletes. This can be attributed to the limited time frame available for participant recruitment and data collection as well. A larger sample would enable a more robust analysis, enhance statistical power, and improve the reliability of effect estimates.

Since we were unable to perform tests twice on separate occasions due to limited accessibility of the participants, it was not possible to provide interday reliability analyses to ensure absence of learning effects. However, the same conditions were consistently applied to both groups, ensuring that any potential learning effects impacted both groups equally. Furthermore, another study limitation is the performance and evaluation of drop jumps with inexperienced children. The DJ is a highly challenging coordinative task to test the reactive strength ability by commonly providing the jumping height, GCT and reactive strength index. However, as focusing on both, maximal jumping height and minimal GCT, in this data collection, faced children with an impossible task and habituation session were not feasible, we focused exclusively on ground contact times, as jumping height was assessed separately for the long SSC using the CMJ. This was also reflected in the introduction of the test, as participants were instructed to perform the DJ with a minimal GCT, while no instructions were given for the jumping height. Another limitation lies in the subjective assessment of the gymnastics tests, even though countermeasures such as video analysis as well as the evaluation by a licensed gymnastics coach were implemented. While these measures significantly minimized inconsistencies in evaluation, a certain degree of subjectivity remains inherent to the assessment process and cannot be entirely eliminated. On top of that, focusing on a single team sport or comparing Parkour to invasive team sports only could yield compelling insights. Considering its cross-sectional design, this study only captures a snapshot of performance differences rather than long-term effects. Future studies should adopt longitudinal approaches to examine whether the observed benefits persist over time and to determine the potential developmental advantages of sustained Parkour training. This is particularly relevant now, as Parkour is increasingly becoming a structured sport in club settings, especially in Germany. However, standardized training plans have yet to be established, with each club currently developing its own approach. This presents a unique opportunity for future research to contribute to evidence-based training guidelines and shape the structured development of Parkour as a competitive and recreational sport. Future studies should investigate both individual sports and intraindividual differences to enhance understanding of traceurs’ physical capabilities and optimize training interventions. Targeting children and adolescents is crucial, given their sensitivity to physical training gains. Furthermore, proprioception tests could be specifically adapted to tracuers’ demands, altered by incorporating tasks with closed eyes (53) or assessing unilateral jumps to identify limb asymmetries (55). Additionally, Parkour training programs may be instrumentalized in interventions to benefit other sports in terms of a rapid and sustainable development of certain functional movements (13, 28) or as a contemporary pedagogical means (5, 25, 27, 29, 56, 57). These limitations must be considered when interpreting results and should be addressed in future research in a developing and promising field of the parcours sports.

## 7 Conclusion

In conclusion, it is emphasized that regular Parkour training induces an array of positive effects on motor performance in children and (pre-)pubertal adolescents. The PG showed better results in the majority of tests (10/13) with statistically significant differences in CMJ, YBT *ant*, CW and PUs. These findings suggest that practicing Parkour at early stages of development can lead to an enhanced overall athletic development, particularly influencing jumping performance, balance, upper body strength and certain gymnastics skills. While this study contributes to our understanding of the physical benefits of practicing Parkour in youth athletes, further longitudinal research is necessary to validate these results. Future studies should aim to confirm or refine these findings by considering training experience, long-term adaptations, and standardized training approaches.

## Data Availability

The raw data supporting the conclusions of this article will be made available by the authors, without undue reservation.

## References

[1] Marchetti PH Luz DA Jr Soares EG Silva FH Uchida MC Felipe L Differences in muscular performance between practitioners and non practitioners of parkour. Int J Sports Sci. (2012) 2(4):36–41. 10.5923/j.sports.20120204.02

[2] Grosprêtre S Lepers R. Performance characteristics of parkour practitioners: who are the traceurs? Eur J Sport Sci. (2015) 16(5):526–35. 10.1080/17461391.2015.1060263 26267256

[3] Angel J. Breaking the Jump. the Secret Story of Parkour’s High Flying Rebellion. Croydon: Quarto Publishing Group (2016).

[4] Puddle DL Maulder PS. Ground reaction forces and loading rates associated with parkour and traditional drop landing techniques. J Sports Sci Med. (2013) 12(1):122–9. 24149735 PMC3761764

[5] Pagnon D Faity G Maldonado G Daout Y Grosprêtre S. What makes parkour unique? A narrative review across miscellaneous academic fields. Sports Med. (2022) 52(1):1029–44. 10.1007/s40279-022-01642-x 35089536

[6] Aggerholm K Larsen SH. Parkour as acrobatics: an existential phenomenological study of movement in parkour. Qual Res Sport. (2017) 9(1):69–86. 10.1080/2159676X.2016.1196387

[7] Larsen SH. What can the parkour craftsmen tell US about bodily expertise and skilled movement? Sport Ethics Phil. (2016) 10(3):1–15. 10.1080/17511321.2016.1217919

[8] Barrera-Domínguez FJ Carmona-Gómez A Tornero-Quiñones I Sáez-Padilla J Sierra-Robles Á Molina-López J. Influence of dynamic balance on jumping-based asymmetries in team sport: a between-sports comparison in basketball and handball athletes. Int J Environ Res Public Health. (2021) 18:1866. 10.3390/ijerph18041866 33672951 PMC7917681

[9] Ramirez-Campillo R García-Hermoso A Moran J Chaabene H Negra Y Scanlan AT. The effects of plyometric jump training on physical fitness attributes in basketball players: a meta-analysis. J Sport Health Sci. (2022) 11(1):656–70. 10.1016/j.jshs.2020.12.005 33359798 PMC9729929

[10] Jakšić D Maričić S Maksimović N Bianco A Sekulić D Foretić N Effects of additional plyometric training on the jump performance of elite male handball players: a systematic review. Int J Environ Res Public Health. (2023) 20:2475. 10.3390/ijerph20032475 36767841 PMC9915565

[11] Cardinale M. Strength training in handball. Aspetar Sports Med J. (2014) 3(1):130–4.

[12] Gerodimos V Manou V Ioakimidis P Perkos S Kellis S. Vertical jumping ability in elite young soccer players. J Human Move Stud. (2006) 51(2):89–102.

[13] Strafford BW van der Steen P Davids K Stone JA. Parkour as a donor sport for athletic development in youth team sports: insights through an ecological dynamics lens. Sports Med Open. (2018) 4:21. 10.1186/s40798-018-0132-5 29797285 PMC5968018

[14] Ball NB Stock CG Scurr JC. Bilateral contact ground reaction forces and contact times during plyometric drop jumping. J Strength Cond Res. (2010) 24(10):2762–9. 10.1519/JSC.0b013e3181cc2408 20613651

[15] Rocha JA da Silva RAD Oliveira MP Szmuchrowski LA Couto BP Drummond MM. Chronic effect of strength training on vertical jump performance in parkour practitioners. Arch Budo Sci Material Arts Extreme Sports. (2017) 13(1):173–8.

[16] Lyons Donegan M Eustace S Morris R Penny R Tallis J. The effects of soccer specific exercise on countermovement jump performance in elite youth soccer players. Children. (2022) 9(12):1861. 10.3390/children9121861 36553305 PMC9777183

[17] Sattler T Hadžić V Dervišević E Markovic G. Vertical jump performance of professional male and female volleyball players: effects of playing position and competition level. J Strength Cond Res. (2015) 29(6):1486–93. 10.1519/JSC.0000000000000781 25436623

[18] Struzik A Pietraszewski B Zawadzki J. Biomechanical analysis of the jump shot in basketball. J Hum Kinet. (2014) 42(1):73–9. 10.2478/hukin-2014-0062 25414741 PMC4234772

[19] Wagner H Finkenzeller T Würth S von Duvillard SP. Individual and team performance in team-handball: a review. J Sports Sci Med. (2014) 13(4):808–16. 25435773 PMC4234950

[20] Kollias I Panoutsakopoulos V Papaiakovou G. Comparing jumping ability among athletes of various sports: vertical drop jumping from 60 centimeters. J Strength Cond Res. (2004) 18(3):546–50. 10.1519/00124278-200408000-00027 15320661

[21] Nasuka N Setiowati A Indrawati F. Power, strength and endurance of volleyball athlete [sic!] among different competition levels. Utop Prax Latinoam. (2020) 25(10):15–23.

[22] Silva DA Petroski EL Gaya AC. Anthropometric and physical fitness differences among Brazilian adolescents who practise different team court sports. J Hum Kinet. (2013) 36(1):77–86. 10.2478/hukin-2013-0008 23717357 PMC3661897

[23] Khan MA Moiz JA Raza S Verma S Shareef MY Anwer S Physical and balance performance following exercise induced muscle damage in male soccer players. J Phys Ther Sci. (2016) 28(10):2942–9. 10.1589/jpts.28.2942 27821967 PMC5088158

[24] Molina-López J Barea Zarzuela I Sáez-Padilla J Tornero-Quiñones I Planells E. Mediation effect of age category on the relationship between body composition and the physical fitness profile in youth handball players. Int J Environ Res Public Health. (2020) 17(7):2350. 10.3390/ijerph17072350 32244283 PMC7177961

[25] Dvorak M Eves N Bunc V Balas J. Effects of parkour training on health-related physical fitness in male adolescents. Open Sports Sci J. (2017) 10:132–40. 10.2174/1875399X01710010132

[26] Chamorro MS Márquez M Borbón OMR Ulate FV. Comparación de los componentes de la aptitud física y compositión corperal en practicantes de parkour según los años de entrenamiento. Rev Movi Humano Salud. (2017) 14(1). 10.15359/mhs.14-1.5

[27] Fernández-Río J Suárez CA. Feasibility and students’ preliminary views on parkour in a group of primary school children. Phys Educ Sport Pedagogy. (2016) 21(3):281–94. 10.1080/17408989.2014.946008

[28] Edriss S. Implementations of tacto software for analysing athletic effects of parkour on youth football player’s performance. LASE J Sport Sci. (2019) 10(1):63–73.

[29] Bernadowski C. Alternative to team sports: the effects of parkour on children. Adolescents and Young Adults: A Case Study. Int J Phys Ed. (2017) 54(4):31–6.

[30] French B Sycamore NJ McGlashan HL Blanchard CCV Holmes NP. Ceiling effects in the movement assessment battery for children-2 (MABC-2) suggest that non-parametric scoring methods are required. PLoS One. (2018) 13(6):e0198426. 10.1371/journal.pone.0198426 29856879 PMC5983505

[31] Marques A Henriques-Neto D Peralta M Martins J Gomes F Popovic S Field-based health-related physical fitness tests in children and adolescents: a systematic review. Front Physiol. (2021) 9:640028. 10.3389/fped.2021.640028 PMC7973114 33748047

[32] Akl AR. A comparison of biomechanical parameters between two methods of countermovement jump. Int J Sports Sci Eng. (2013) 7(2):123–8.

[33] Scinicarelli G Trofenik M Froböse I Wilke C. The reliability of common functional performance tests within an experimental test battery for the lower extremities. Sports. (2021) 9(7):100. 10.3390/sports9070100 34357934 PMC8309832

[34] Boyer C Tremblay M Saunders TJ McFarlane A Borghese M Lloyd M Feasibility, validity and reliability of the plank isometric hold as a field-based assessment of torso muscular endurance for children 8 to 12 years of age. Pediatr Exerc Sci. (2013) 25(3):407–22. 10.1123/pes.25.3.407 23877226

[35] Donham-Foutch S. Teaching skills and health-related fitness through a preservice gymnastics program. J Phys Educ Recreat Dance. (2007) 78(5):35–44. 10.1080/07303084.2007.10598022

[36] Gerling IE. Basisbuch Gerätturnen. Von Bewegungsgrundformen mit Spiel und Spaß zu Basisfertigkeiten. Aachen: Meyer & Meyer (2014).

[37] Whiteley R. Coach’s eye. Br J Sports Med. (2015) 49(20):1349. 10.1136/bjsports-2015-094656 26048895

[38] Mkaouer B Hammoudi-Nassib S Amara S Chaabène H. Evaluating the physical and basic gymnastics skills assessment for talent identification in men’s artistic gymnastics proposed by the international gymnastics federation. Biol Sport. (2018) 35(4):383–92. 10.5114/biolsport.2018.78059 30765924 PMC6358534

[39] Vernetta M Montosa I Beas-Jiménez J López-Bedoya J. Batería funcional ARISTO en gimnasia rítmica: protocolo de test específicos para la evaluación de jóvenes gimnastas en un Ámbito de entrenamiento saludable. Rev Andaluza Med Deporte. (2017) 10(3):112–9. 10.1016/j.ramd.2017.02.001

[40] Mamuti F Rexhepi F. Kinematic analysis of handstand in gymnastics – educational gymnastics. Int J Sport Sciences Health. (2018) 5(9):85–91.

[41] Maldonado G Soueres P Watier B. Strategies of parkour practitioners for executing soft precision landings. J Sports Sci. (2018) 36(22):2551–7. 10.1080/02640414.2018.1469226 29690830

[42] Standing JR Maulder PS. A comparison of the habitual landing strategies from differing drop heights of parkour practitioners (traceurs) and recreationally trained individuals. J Sports Sci Med. (2015) 14(4):723–31. 26664268 PMC4657414

[43] You C Huang C. Effects of leg stiffness regulated by different landing styles on vertical drop jump performance. J Hum Kinet. (2022) 83(1):29–37. 10.2478/hukin-2022-0066 36157958 PMC9465758

[44] Flanagan E Comyns TM. The use of contact time and the reactive strength Index to optimize fast stretch-shortening cycle training. Strength Cond J. (2008) 30(5):32–8. 10.1519/SSC.0b013e318187e25b

[45] Clegg JL. An existential phenomenological examination of parkour and freerunning (Doctoral dissertation). San Jose State University (2011).

[46] Nyman E. Biomechanics of gymnastics. In: Sweeney E, editor. Gymnastics Medicine. Cham: Springer (2020). p. 27–54.

[47] Hobara H Inoue K Kobayashi Y Ogata T. A comparison of computation methods for leg stiffness during hopping. J Appl Biomech. (2014) 30(1):154–9. 10.1123/jab.2012-0285 24676522

[48] Laffaye G Choukou MA Benguigui N Padulo J. Age- and gender-related development of stretch shortening cycle during a sub-maximal hopping task. Biol Sport. (2016) 33(1):29–35. 10.5604/20831862.1180169 26985131 PMC4786579

[49] Meylan C McMaster T Cronin J Mohammad NI Rogers C Deklerk M. Single-leg lateral, horizontal, and vertical jump assessment: reliability, interrelationships, and ability to predict sprint and change-of-direction performance. J Strength Cond Res. (2009) 23(4):1140–7. 10.1519/JSC.0b013e318190f9c2 19528866

[50] John C Rahlf AL Hamacher D Zech A. Influence of biological maturity on static and dynamic postural control among male youth soccer players. Gait Posture. (2019) 68:18–22. 10.1016/j.gaitpost.2018.10.036 30439683

[51] Read PJ Oliver JL Myer GD Farooq A De Ste Croix M Lloyd RS. Utility of the anterior reach Y-BALANCE test as an injury risk screening tool in elite male youth soccer players. Phys Ther Sport. (2020) 45:103–10. 10.1016/j.ptsp.2020.06.002 32726731 PMC9892799

[52] Meokahar F Yoga M. Phenomenology of the parkour practitioner. 3rd Jogjakarta Communication Conference (JCC 2021); Atlantis Press (2021).

[53] Seyhan S. Comparison of physical and physiological performance features of parkour and gymnastics athletes. J Ed Learn. (2019) 8(2):111–6. 10.5539/jel.v8n2p111

[54] Rabaglietti E Mulasso A Arzenton M. Parkour vs artistic gymnastics among pre-adolescents: a multidimensional study on the psychological adjustment in the [sic!] sport activities. Adv Phys Ed. (2021) 11(1):47–60. 10.4236/ape.2021.111004

[55] Grosprêtre S Khattabi SE. Training habits and lower limb injury prevention in parkour practitioners. Mov Sport Sci Sci Motricité. (2022) 115:43–55. 10.1051/sm/2021024

[56] Juan DF García-Martínez S Martínez VJS Ferriz-Valero A. Application of parkour in physical education: agility learning and improvement. J Phys Ed Sport. (2022) 22(3):709–14. 10.7752/jpes.2022.03089

[57] Naschold I. Parkour macht schule: sportliches angebot begeistert schüler wie profis. Int Fachzeitschrift Sportstätten Freizeitanlagen. (2019) 53(4):42–42.

