# Supplementary material for: Comparison of basic motor skills and physical fitness between (pre-)pubertal children from parkour and team sports
Source: Front Sports Act Living. 2025 Mar 27;7:1562561. doi: 10.3389/fspor.2025.1562561 (PMC11983557; doi:10.3389/fspor.2025.1562561)
Supplement: Supplementary file 1 [file Datasheet1.pdf]

Supplementary Material

FIGURE 1 | Test battery

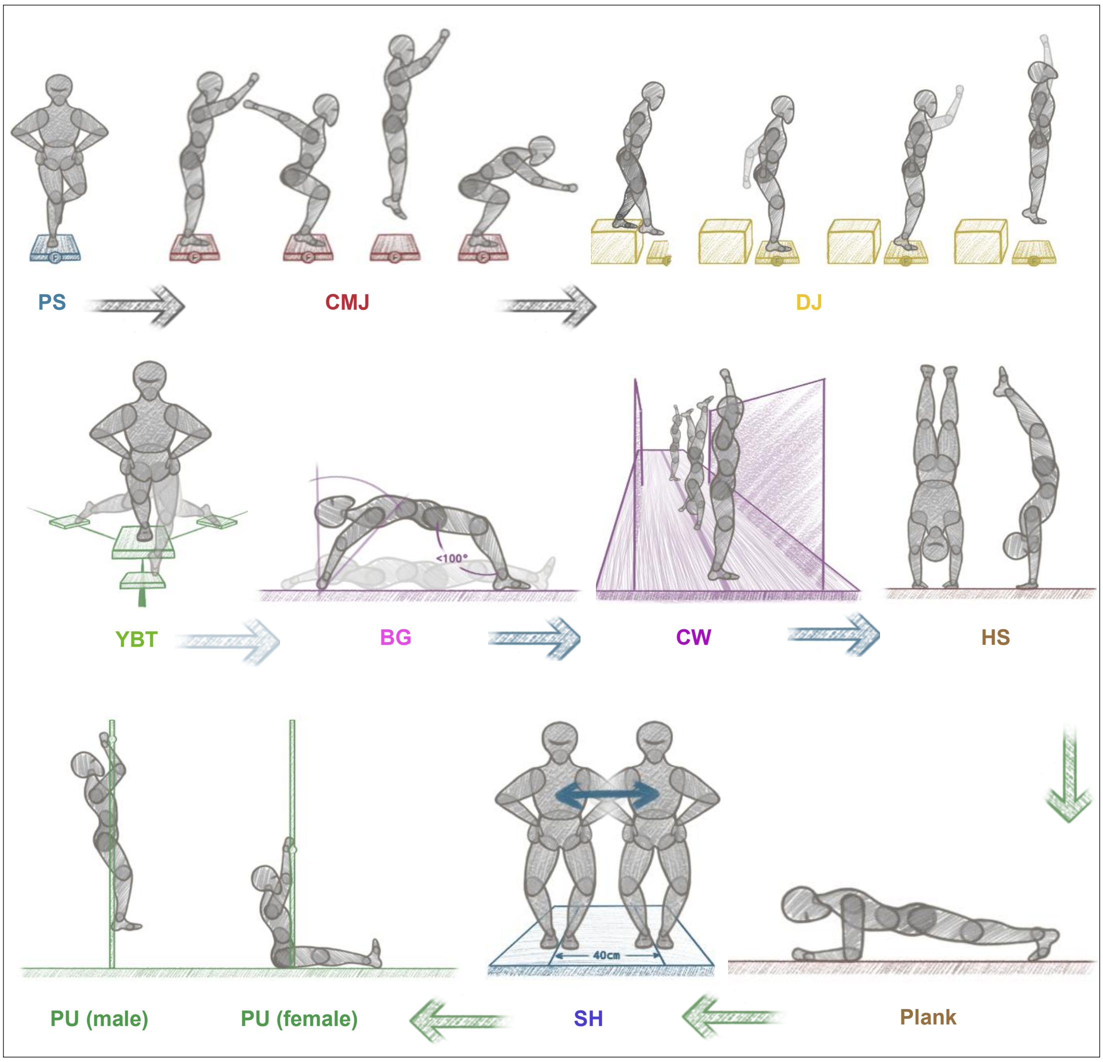

FIGURE 2 | Push-ups (Mean ± SD)

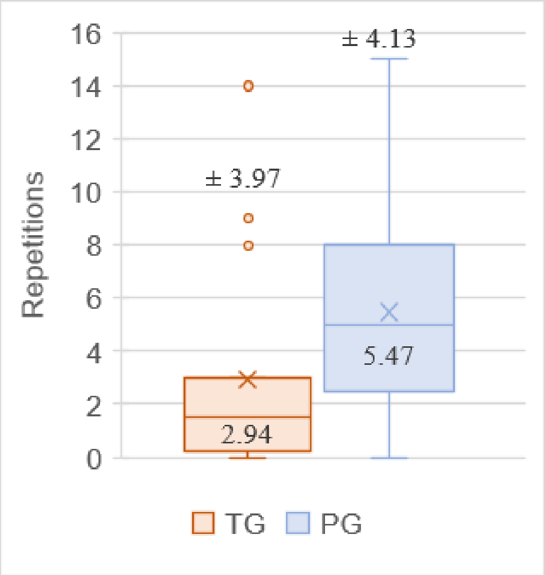

Note: The orange dots represent outliers (above 1.5 IQR) which were only observed in this specific instance.
